# Endoscopic Ultrasound Guided Gastro-Gastrostomy for Management of Pouch Outlet Obstruction Secondary to Vertical Banded Gastroplasty: Efficacy and Safety

**DOI:** 10.1007/s11695-025-07957-8

**Published:** 2025-07-16

**Authors:** Gilles Hoilat, Melis Celdir, Henning Gerke

**Affiliations:** 1 Gastroenterology and Hepatology, University of Iowa Hospital and Clinics, Iowa City, USA; 2 Present Address: Gastroenterology and Hepatology, MercyOne-Genesis, Davenport, USA

**Keywords:** VBG, Outlet obstruction, EUS, Gastrogastrostomy

## Abstract

**Background:**

Vertical banded gastroplasty (VBG) was one of the preferred bariatric procedures in the 1980s. It includes a vertical staple line to create a small stomach pouch separated from the remainder of the stomach by a dime size hole. Symptoms of pouch outlet obstruction are a common late complication. At our center, we adopted an EUS-guided gastrogastrostomy with a lumen-apposing metal stent to treat this.

**Methods:**

We retrospectively analyzed nine consecutive patients who underwent EUS-guided gastrogastrostomy for pouch outlet obstruction (POO) secondary to vertical banded gastroplasty between June 2019 and January 2024.

**Results:**

The follow-up period varied with a median period of 5.5 months (IQR 5–21). Patients with obstructive symptoms reported resolution of their symptoms and were able to tolerate a general diet. The median weight change was + 3.1 kg (IQR 1–8.45) with a median relative difference of + 3.7% (IQR 0.84–7.25). No adverse events were encountered, except for late stent migration in two patients.

**Conclusions:**

Gastrogastrostomy with LAMS is effective and safe in treating pouch outlet obstruction in patients with a VBG anatomy. However, late stent migration may occur if not removed. Follow-up is required to monitor for fistula persistence after stent removal and to assess weight gain.

## Methods

We conducted a retrospective study analyzing the outcomes of patients who underwent EUS-gastrogastrostomy for POO related to VBG in our tertiary referral center between June 2019 and January 2024. The diagnosis of POO was based on symptoms including nausea, regurgitation, intractable heartburn, intolerance to a regular diet, and weight loss.

**Fig. 1:**
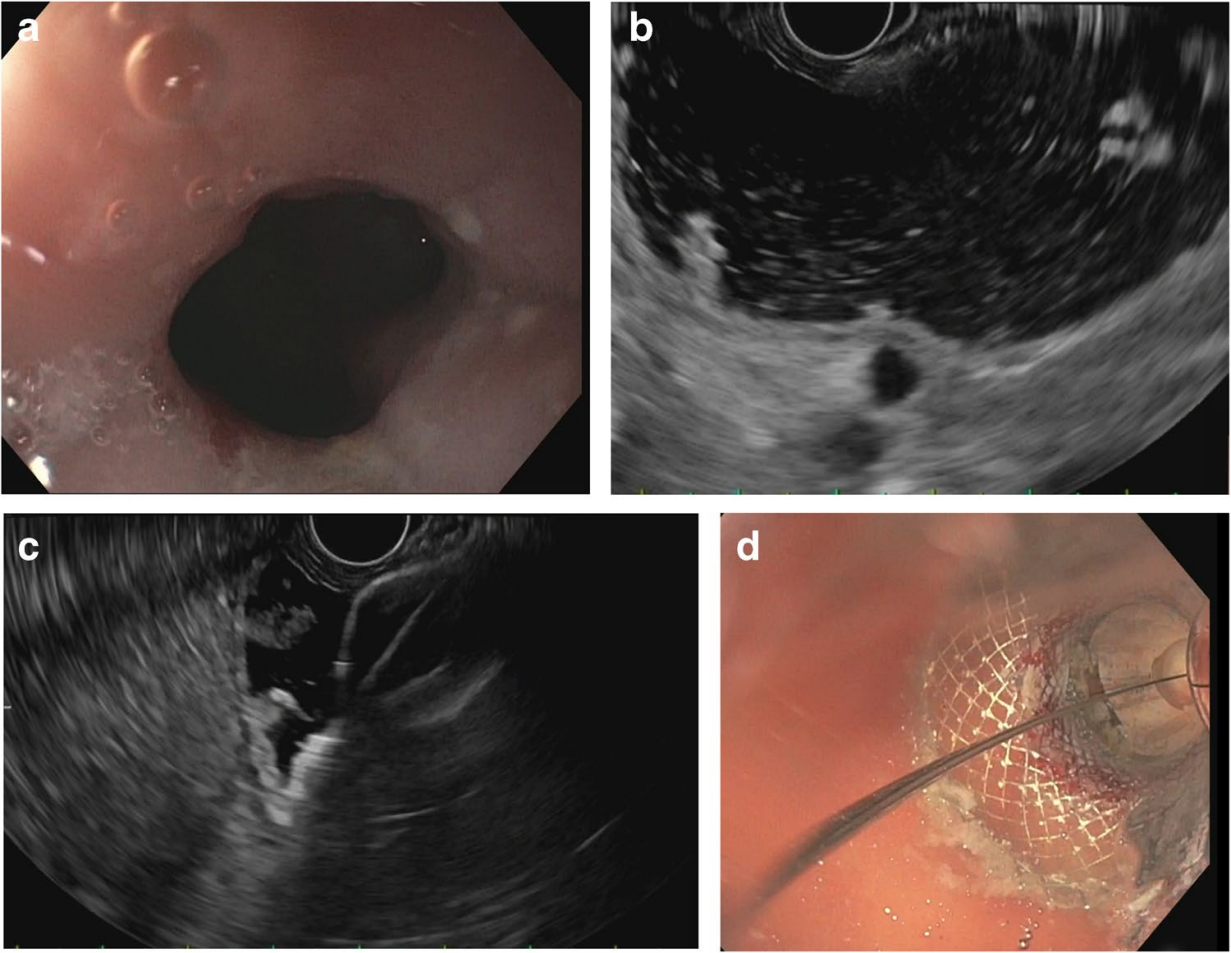
**a** Narrow pouch outlet. **b** EUS showing water filled fundus. **c** Hot AXIOS inserted with internal flange being deployed. **d** Stent being dilated with an 18-mm balloon dilator through the scope

**Fig. 2:**
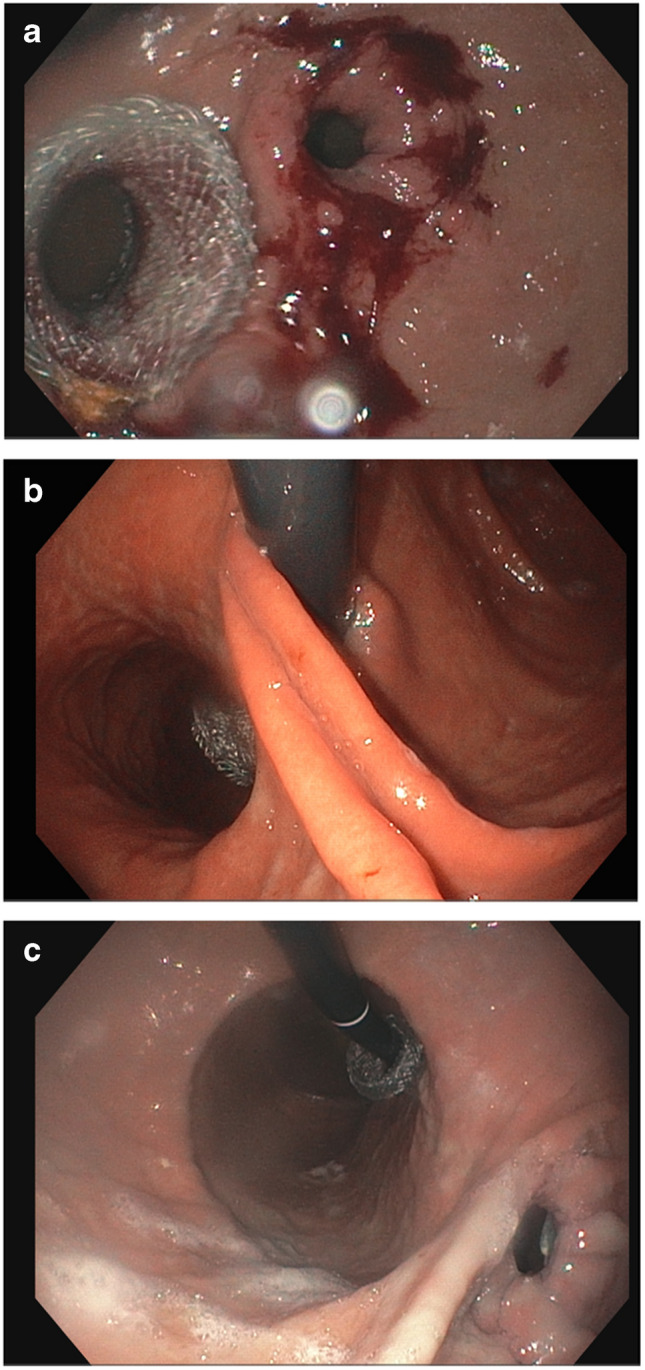
**a** Forward EGD view with pouch outlet and proximal stent flange in the gastric pouch. **b** Retroflexed view through the pouch outlet looking at the distal stent flange in stomach proper. **c** Retroflexed view through the stent looking at the pouch outlet

The EUS-gastrogastrostomy procedures were done under general anesthesia. Initially, the anatomy was assessed with a gastroscope (GIF-H190, Olympus, Center Valley, PA, USA) and retained food was removed from the gastric pouch if present (Fig. 1a). The gastric fundus was then filled with water (Fig. 1b). A gastrogastrostomy was created from the pouch to the fundus with a LAMS (Hot Axios™, 20 mm × 10 mm, Boston-Scientific, Marlborough, MA, USA) under EUS guidance with a therapeutic linear echoendoscope (GF-UCT180, Olympus) using cautery (ESG 100 Olympus, cut 1, 120 watts), except in one patient where the stent was placed without cautery through a small, spontaneous gastrogastric fistula (Fig. 1c). The stent lumen was dilated to 18 mm using a through-the-scope balloon (Fig. 1d). The stent was traversed with the gastroscope to confirm adequate position (Fig. 2a–c).

Follow-up was obtained by chart review and telephone encounters.

## Results

The patient cohort consisted of nine patients, seven females (77.8%) and two males (22.2%), with a mean age of 69.7 ± 10.2 years (range 48–81). Mean weight at clinical presentation was 101.5 ± 18.9 kg (range 68.3–134.6) with a mean BMI of 34.75 ± 5.2 (range 25.7–42). The time passed since the VBG surgery was 40.4 ± 7.1 years (range 28–50). Symptoms at presentation were nausea in three (33.3%) vomiting in six (66.7%), regurgitation in four (44.4%), heartburn in four (44.4%), dysphagia in two patients (22.2%), and shortness of breath that was attributed to recurrent aspiration in one patient (11.1%). Five patients (55.6%) had more than one symptom (Table 1).

**Table 1:** Patient demographics of individuals undergoing endoscopic ultrasound-guided gastrogastrostomy for management of gastric outlet stenosis secondary to vertical banded gastroplasty

Case	Age	Gender	Weight (kg)	BMI	Date of VBG procedure	Time since VBG (yr)	Symptoms on presentation					
							Nausea	Vomiting	Regurgitation	Heartburn	Dysphagia	Shortness of breath (aspiration)
1	78	Female	99.4	35	1986	37	Yes	Yes	Yes	No	No	No
2	72	Male	106.2	33.5	1970	50	No	Yes	No	Yes	Yes	No
3	60	Female	97.4	31.9	1992	32	No	No	Yes	Yes	No	No
4	72	Female	107.3	42	1980	43	No	Yes	Yes	No	No	No
5	67	Male	134.6	41.5	1985	39	No	No	No	No	No	Yes
6	81	Female	94.4	35.7	1976	45	Yes	No	No	No	Yes	No
7	73	Female	86.9	32.7	1979	44	Yes	Yes	No	No	No	No
8	76	Female	68.3	25.7	1978	46	No	Yes	No	Yes	No	No
9	48	Female	119.4	38.97	1996	28	No	Yes	Yes	Yes	No	No

The median procedure duration was 39 min (IQR 31.5–61.5). In one patient, the procedure was done during hospitalization for recurrent aspiration pneumonias; all other patients were outpatients, and none of these was admitted to the hospital following the procedure nor within 30 days of the procedure.

One patient was lost to follow-up. Of the other eight patients, the median follow-up period was 5.5 months (IQR 5–21). All eight patients reported resolution of symptoms and were able to tolerate a general diet. The median weight change was + 3.1 kg (IQR 1–8.45) with a median relative difference of + 3.7% (IQR 0.84–7.25). Weight gain occurred in seven of eight patients. One patient lost weight after late stent migration that was complicated by small bowel obstruction.

Late stent migration occurred in two of eight patients (25%): in one patient, the stent migrated into the stomach 3 years post-procedure and was endoscopically removed. In another patient, the stent migrated into the small bowel 8 months post-procedure, necessitating an exploratory laparotomy and small bowel enterotomy. The LAMS in this patient had been placed into an existing gastrogastric fistula, possibly resulting in the inferior anchoring. No other adverse events were encountered (Table 2).

**Table 2:** Patient outcomes after endoscopic ultrasound-guided gastrogastrostomy for management of gastric outlet obstruction secondary to vertical banded gastroplasty

Case	Procedure duration (min)	Hospital stay (days)	Follow-up (months)	Weight at follow-up (kg)	∆Weight (kg)/∆%	BMI	Long-term complications
1	34	0	6	102.9	+ 3.5 kg/+ 3.52%	36.6	None
2	78	0	48	90	− 16.2 kg/− 15.2%	28.4	Stent migrated 3 years post-procedure into stomach, which was endoscopically removed
3	39	0	4	103	+ 5.6 kg/+ 5.75%	36.6	None
4	35	0	6	107.5	+ 0.2 kg/+ 0.18%	42	Sent migrated 8 months post-procedure into small bowel, status post exploratory laparotomy, small bowel enterotomy, and removal of axios stent
5	83	0	5	146.4	+ 11.8 kg/+ 8.76%	45	None
6	45	0	36	105.7	+ 11.3 kg/+ 12%	40	None
7	36	0	NA	NA	NA	NA	NA
8	36	0	5	71	+ 2.7/+ 3.95%	26.7	None
9	42	0	5	121.2	+ 1.8/+ 1.50%	39.4	None

∆Weight kg (difference between weight in kilograms at follow-up and weight in kilograms at presentation in absolute number)

**∆**% (percentage of weight change between weight at follow-up and weight at presentation)

*BMI* body mass index

## Discussion and Conclusion

The principle of vertical gastroplasty is to create a small gastric pouch with delayed food passage into the remainder of the stomach. Unfortunately, the intended food stasis does not address the underlying metabolic mechanisms of appetite and satiety, and patients commonly regain most of the initially lost weight. Further, impaired emptying of the pouch leads to excessive symptoms resembling gastric outlet obstruction in many patients.

Revisional bariatric surgery is often technically challenging and carries a high risk of complications in these mostly elderly patients [1]. Endoscopic attempts to widen the pouch outlet with balloon dilation, steroid injection, incisional therapy, and placement of stents have been described in the literature [2] but are prone to fail because of the nonstretchable ring or mesh band which stabilizes the outlet. Recent advances in endoscopic stent technology, specifically the development of LAMS, have facilitated the creation of luminal connections. Gastrogastrostomies using lumen-apposing metal stents are already commonly performed in patients with Roux-en-Y gastric bypass anatomy who require an ERCP, and it is intriguing to use the same technology to bypass the narrow pouch outlet in patients with VBG. This has been described in two case reports. In both cases [3, 4], the endoscopic technique that was used was similar to our method, but no long-term follow-up was available. Our series of nine patients with clinical follow-up in eight confirms that EUS-gastrogastrostomy is highly successful in resolving symptoms of pouch outlet obstruction. However, late stent migration occurred in two of eight patients. While we initially intended to keep the stents in place indefinitely to prevent spontaneous closure of the gastrostomy, the occurrence of stent migration lead us to change our protocol to include elective stent removal. Currently, it is unknown if resolution of symptoms is durable after stent removal. A multicenter US study on long-term follow-up and fistula closure in patients undergoing endoscopic ultrasound-directed trans-gastric ERCP (EDGE-procedure) [5] showed that the only variable that predicted fistula persistence was total LAMS indwell time. The total duration of time LAMS was in place was 86 days in the persistent group, compared to 50 days in the nonpersistent group (*P* < 0.004). Assuming that these results can be transferred to gastrogastrostomies in VBG patients, one can be hopeful that tract patency is durable if stents are left in place for 3–6 months.

In summary, EUS-guided gastrogastrostomy appears to be a safe and highly effective treatment for patients with impaired pouch emptying after VBG. However, late stent migration may occur if stents are not electively removed. Follow-up is required to monitor for fistula persistence after stent removal and to assess weight gain.

## Data Availability

No datasets were generated or analysed during the current study.
